# GSK3**β** and the aging kidney

**DOI:** 10.1172/JCI155885

**Published:** 2022-02-15

**Authors:** Jordan A. Kreidberg, Valerie A. Schumacher

**Affiliations:** Departments of Urology and Pediatrics, Boston Children’s Hospital and Departments of Surgery and Pediatrics, Harvard Medical School, Boston, Massachusetts, USA.

## Abstract

Kidney function decreases with age and may soon limit millions of lives as the proportion of the population over 70 years of age increases. Glycogen synthase kinase 3**β** (GSK3**β**) is involved with metabolism and may have a role in kidney senescence, positioning it as a target for complications from chronic kidney disease. However, different studies suggest GSK3 has contrasting effects. In this issue of the *JCI*, Fang et al. explored the function of GSK3**β** and the interplay with lithium using human tissue and mouse models. Notably, GSK3**β** was overexpressed and activated in aging mice, and depleting GSK3**β** reduced senescence and glomerular aging. In this Commentary, we explore the similarities and differences between Fang et al. and previous findings by Hurcombe et al. These findings should prompt further study of lithium and other GSK3**β** inhibitors as a means of extending glomerular function in individuals with chronic kidney disease.

## GSK3 affects diverse processes

The population of the world is aging and it is estimated that, over the next half a century, as much as 30% of the population may be in their seventh decade. It is also well established that kidney function decreases with age. Many studies have shown this decrease in kidney function to be manifested by a decrease in kidney size (1, 2) as well as decreased glomerular filtration rate (GFR) (3, 4). Therefore, a substantial portion of the population may have GFRs in a range indicative of chronic kidney disease (3, 4). As kidney disease does not become apparent until there is a remarkable loss of kidney function, there are tens of millions of individuals with some degree of chronic kidney disease. Histological studies have shown that the percentage of glomeruli showing signs typical of glomerulosclerosis increases with age (4). Cellular senescence has a central role in the aging process and has been studied intensively. The major molecular pathways involved in cellular senescence appear to be those regulated by p53, p16INK4A, and downstream cyclin-dependent-kinase inhibitors (5). Wnt signaling also likely has a role in the aging process (6).

GSK3 is an enzyme that has two highly conserved isoforms, GSK3α and GSK3β (7). As indicated by its name, GSK was originally identified as a regulator of glucose metabolism, acting downstream of insulin (8). Active GSK3 phosphorylates glycogen synthase, decreasing its activity and decreasing the storage of glucose as glycogen. Insulin signaling via phosphoinositide 3-kinase and AKT inactivates GSK3, increasing the activity of glycogen synthase and the storage of glucose as glycogen. Thus, GSK enzymatic activity is crucial in regulating glucose levels. Is it surprising that an enzyme of such critical importance in energy metabolism should have such a pleiotropic role in regulating such a diverse array of biological pathways? Perhaps this pleiotropy is an example of evolutionary efficiency. GSK3β, the isoform that has received greater study, is probably best known, beyond its role in regulating glycogen synthesis, for its ability to phosphorylate β-catenin, targeting it for proteasomal degradation, thereby suppressing canonical Wnt signaling. Indeed, for many years the most commonly accepted approach to boosting canonical Wnt signaling has involved the use of GSK3β inhibitors, originally lithium (9), and more recently small molecules such as CHIR99021 (10). However, the enzymatic activity of GSK3β is able to phosphorylate serines and threonines on a wide range of proteins, such that GSK3β activity may have pleiotropic effects on cell physiology and particularly on cell senescence. These targets include p53, PTEN, cyclin D1, HIF-1α, CREB, NFAT transcription factors, SMAD1, c-Jun, FAK, and others. Phosphorylation by GSK3β can activate or inhibit its substrate, depending on the target (7). It should also be acknowledged that GSK3β itself has several serine, threonine, and tyrosine phosphorylation sites and is a substrate of various kinases including p90RSK, p70S6K, AKT, PKA, ERK, FYN, and p38 MAPK. Thus, GSK3β is often considered a nexus through which many upstream and downstream signaling pathways are integrated (7). For this reason and others, the role of GSK3β has been studied in a multitude of biological systems, from stem cell renewal to cellular senescence in almost every organ system, and with regard to its role in a broad range of human disease.

## GSK3β activity and podocyte function

Past studies had suggested that activation of β-catenin was detrimental to podocytes (reviewed in ref. 11). These findings raise the question of whether changes in GSK3β activity may affect podocyte function, possibly by altering the levels of canonical Wnt signaling. Indeed, the role of GSK3β in various types of kidney ailments, including glomerular disease and polycystic kidney disease, has received serious attention in the past several years. Several studies have suggested disparate effects of inhibiting or genetically inactivating GSK3β for preserving podocyte function (12–14).

The recent studies by Hurcombe et al. and Fang et al. add to our understanding of the role of GSK in glomerular disease (15, 16). Both studies used pharmacological treatments and targeted genes in podocytes. However, the findings have, to some extent, led researchers to opposing conclusions, most likely because of important differences in their respective experimental approaches. Hurcombe et al. studied mice with podocyte-specific inactivation of both GSK3α and GSK3β (15). The researchers claimed that neither single knockout led to any apparent phenotype after 2 years of observation. However, using a constitutively active Cre recombinase construct expressed specifically in podocytes, loss of both GSK3α and GSK3β was incompatible with podocyte function and there was widespread effacement (loss of podocyte foot process architecture), and none of the mutant mice survived beyond about 15 days. A similar result was obtained studying nephrocytes in mutant Drosophila. Hurcombe et al. also performed an inducible postnatal podocyte-specific knockout of both GSK3α and GSK3β (referred to as DKO mice), and nearly all DKO mice developed albuminuria and glomerulosclerosis. Further, lithium-treated Wistar rats developed proteinuria. In a surprising turn, Hurcombe et al. found that even though an increase in activated (nonphosphorylated) β-catenin was observed in their DKO mice, genetic ablation of β-catenin along with both isoforms of GSK3 failed to rescue the GSK3 DKO phenotype, casting doubt on the hypothesis that podocyte dysfunction in their DKO mice was primarily due to excessive activation of β-catenin and canonical Wnt signaling. Further studies by Hurcombe et al. suggest that podocytes in DKO mice were attempting to reenter the cell cycle. As podocytes are terminally differentiated cells, pathological situations involving podocyte reentry into the cell cycle leads to their loss and the onset of albuminuria. Hurcombe et al. also found a disruption of HIPPO signaling in podocytes of DKO mice and increased expression of YAP/TAZ target genes.

**Figure 1:**
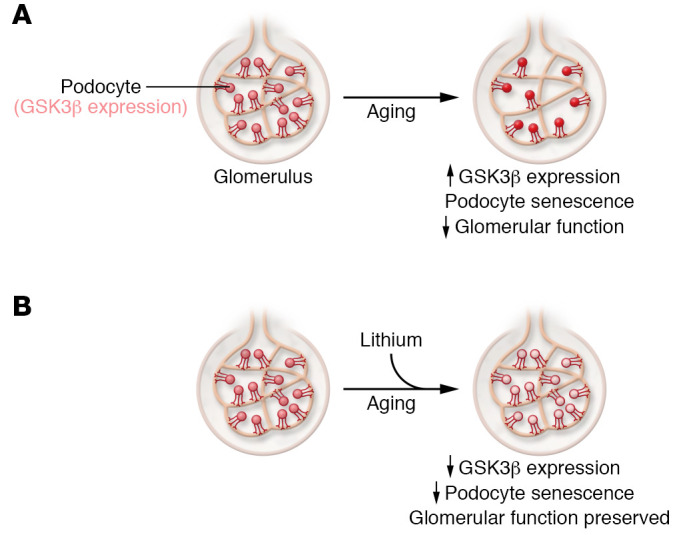
A model for the effects of low-dose lithium on kidney function. With aging, GSK3β expression increases, leading to a reduction in podocytes within glomeruli. Low-dose lithium blocks increased GSK3β expression to reduce senescence, mitigate podocyte loss, and preserve glomerular function (16).

In this issue of the *JCI*, Fang et al. present an alternate view of how GSK3 may affect podocytes, and relate their findings to cellular senescence and glomerular aging in humans (ref. 16 and Figure 1). A difference between the studies of Hurcombe et al. and Fang et al. is that Hurcombe et al. studied mice in which both isoforms of GSK3 had been inactivated, whereas Fang et al. focused exclusively on GSK3β in human tissue and mice. Fang and colleagues initially found that GSK3β expression was elevated in normal human kidney tissue obtained from individuals in a 46- to 60-year-old age group, compared with those younger than age 46. Moreover, GSK3β levels correlated with expression of p16INK4A and inversely correlated with podocyte number. Similar observations were made in aged mice. Fang et al. went on to genetically inactivate GSK3β in mice and observed that depleting GSKβ mitigated podocyte loss and age-related albuminuria. Using immortalized podocytes, Fang et al. also demonstrated a physical interaction between GSK3β and both p16INK4A and p53, and that GSK3β regulated the phosphorylation of p16INK4A and p53, further supporting a role for age-related elevation of GSK3β expression in cellular senescence. In contrast to the observations of Hurcombe et al., Fang et al. observed that once-weekly dosing of mice with lithium decreased cellular senescence as measured by p16INK4A and p53 expression, and preserved age-related glomerular function as measured by albumin/creatine ratio and serum creatinine levels. Finally, Fang and researchers used urine specimens from humans undergoing long-term lithium treatment for psychiatric disease, finding decreased levels of p16INK4A in WT-1^+^ cells, representing exfoliated podocytes, compared with urine from non–lithium-treated individuals (16).

## A simple approach

Are the results of Hurcombe et al. (15) and Fang et al. (16) contradictory? Not necessarily, or at least not entirely. As mentioned above, Hurcombe et al. demonstrated that GSK3 activity is required in podocytes, whereas Fang et al. primarily studied increased expression of GSK3β in aging mice. The findings of Hurcombe et al. strongly suggest some redundancy of function between GSK3α and GSK3β in podocytes (15). Where the studies differ is that Hurcombe et al. reported no phenotype in either single-mutant strains of mice, whereas Fang et al. found improved function in the single GSK3β knockout. It is unclear whether Hurcombe et al. examined their single-knockout mice for improved function, as opposed to loss-of-function phenotypes. Additionally, glomerular disease phenotypes are well known to show dramatic differences between different strains of inbred mice, for reasons that are incompletely understood. The studies of Hurcombe et al. and Fang et al. also found opposing results with lithium-treated animals, the former using rats and the latter mice (15, 16). Previous studies in humans have documented lithium-related nephrotoxicity, involving the glomerulus and tubulointerstitial components (17, 18). Other studies have noted some beneficial effects of lithium (reviewed in ref. 19). Thus, the role of lithium in kidney disease remains incompletely understood. While the studies discussed here attempted to duplicate lithium levels in humans treated for psychiatric disease, it is still possible that differences in experimental design, including the route of administration and dosing, yielded the differing results. The studies of Hurcombe et al. and Fang et al. demonstrate that GSK3 provides essential signaling in podocytes, and that increased expression of GSK3β, in particular, may explain why kidney function declines with age (15, 16). Declining kidney function will be an increasingly crucial determinant of public health, costing society billions of dollars over the next generation. Therefore, the studies of Fang et al. may provide a potentially simple and inexpensive approach to preserving kidney function (16).

## Version 1 02/15/2022

Electronic publication
